# Do Galactolipid Synthases Play a Key Role in the Biogenesis of Chloroplast Membranes of Higher Plants?

**DOI:** 10.3389/fpls.2018.00126

**Published:** 2018-02-08

**Authors:** Joana Rocha, Milène Nitenberg, Agnès Girard-Egrot, Juliette Jouhet, Eric Maréchal, Maryse A. Block, Christelle Breton

**Affiliations:** ^1^Université Grenoble Alpes, Grenoble, France; ^2^CERMAV, CNRS, Grenoble, France; ^3^GEMBAS Team, ICBMS, UMR 5246 CNRS, University of Lyon, Lyon, France; ^4^LPCV, UMR 5168 CNRS/CEA/INRA/UGA, Université Grenoble Alpes, Grenoble, France

**Keywords:** galactolipids, MGDG, DGDG, chloroplast, biosynthesis, Arabidopsis

## Abstract

A unique feature of chloroplasts is their high content of the galactolipids monogalactosyldiacylglycerol (MGDG) and digalactosyldiacylglycerol (DGDG), which constitute up to 80% of their lipids. These galactolipids are synthesized in the chloroplast envelope membrane through the concerted action of galactosyltransferases, the so-called ‘MGDG synthases (MGDs)’ and ‘DGDG synthases (DGDs),’ which use uridine diphosphate (UDP)-galactose as donor. In Arabidopsis leaves, under standard conditions, the enzymes MGD1 and DGD1 provide the bulk of galactolipids, necessary for the massive expansion of thylakoid membranes. Under phosphate limited conditions, plants activate another pathway involving MGD2/MGD3 and DGD2 to provide additional DGDG that is exported to extraplastidial membranes where they partly replace phospholipids, a phosphate-saving mechanism in plants. A third enzyme system, which relies on the UDP-Gal-independent GGGT (also called SFR2 for SENSITIVE TO FREEZING 2), can be activated in response to a freezing stress. The biosynthesis of galactolipids by these multiple enzyme sets must be tightly regulated to meet the cellular demand in response to changing environmental conditions. The cooperation between MGD and DGD enzymes with a possible substrate channeling from diacylglycerol to MGDG and DGDG is supported by biochemical and biophysical studies and mutant analyses reviewed herein. The fine-tuning of MGDG to DGDG ratio, which allows the reversible transition from the hexagonal II to lamellar α phase of the lipid bilayer, could be a key factor in thylakoid biogenesis.

## Introduction

**FIGURE 1:**
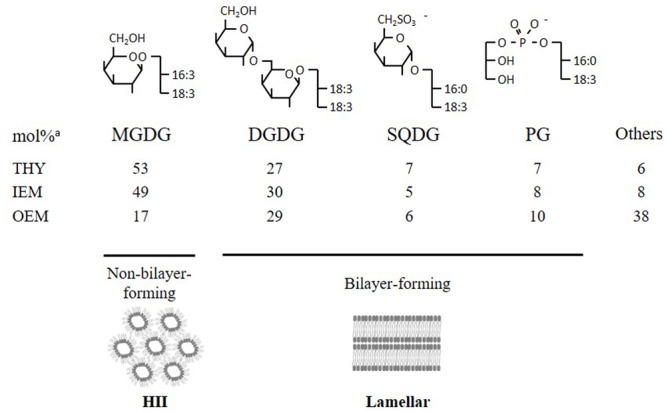
Structures of the conserved quartet of lipids and their abundance (expressed in mol%) in chloroplast membranes. The small polar head of MGDG resembles a truncated cone, which induces a negative curvature favoring its organization into inverted micelles (HII phase). DGDG, SQDG, and PG form lamellar phases. THY, thylakoids; IEM, inner envelope membrane; OEM, outer envelope membrane. ^a^From Block et al., 1983.

Photosynthetic membranes, or thylakoids, have a unique lipid composition that has been remarkably conserved from cyanobacteria to chloroplast-containing eukaryotes (**Figure 1**). The bulk of lipids is composed of the non-phosphorous and uncharged galactoglycerolipids monogalactosyldiacylglycerol (MGDG) and digalactosyldiacylglycerol (DGDG), which represent up to 80% of total lipids (Block et al., 1983). Other lipids mostly consist of the anionic sulfoquinovosyl diacylglycerol (SQDG) and phosphatidylglycerol (PG). MGDG is exclusively found in plastids, but DGDG can also be found in non-plastidial membranes, under specific conditions such as phosphate (Pi) deprivation, where it can substitute phospholipids (Härtel et al., 2000; Jouhet et al., 2004; Andersson et al., 2005). Galactolipids are believed to play essential roles in the light-dependent conversion of prolamellar bodies to thylakoid membranes in germinating seeds and in the dynamic organization of highly stacked grana membranes and photosynthetic machinery in response to light variations (Fujii et al., 2017; Gabruk et al., 2017). MGDG and DGDG are also known to stabilize the photosystem protein complexes in chloroplasts (Jones, 2007; Mizusawa and Wada, 2012; Kobayashi, 2016).

The MGDG and DGDG have also a major role in determining the physicochemical properties of thylakoid membranes. MGDG, which accounts for half of the total lipids, is a ‘non-bilayer forming lipid’ due to its cone-like shape, whereas the three other major components DGDG, SQDG, and PG are ‘bilayer-forming lipids’ (**Figure 1**). Maintaining a constant MGDG/DGDG ratio in thylakoid membranes (at least under standard growth conditions) seems crucial for the stability and functional integrity of photosynthetic membranes (Dörmann and Benning, 2002).

The efficiency of light energy capture and its conversion relies on the rapid expansion of thylakoids, thus requiring an effective and efficient system for providing the bulk of galactolipids. In Arabidopsis, the four major lipid components of photosynthetic membranes are synthesized in the chloroplast envelope (Boudière et al., 2014; Kobayashi, 2016). DAG is the direct precursor for galactolipid formation and it can be produced within the chloroplast via the ‘prokaryotic’ pathway or from extra-plastidial phosphatidylcholine (PC) via the ‘eukaryotic’ pathway in the ER (Siebertz et al., 1979; Browse et al., 1986). The flux of DAG must therefore be tightly controlled to meet the dramatic demand of the chloroplast without adversely impacting the needs of other cell membranes.

Although galactolipids represent the major lipid components of oxygenic photosynthetic organisms, their biosynthesis differs between cyanobacteria and plants (Awai, 2016). In plants, MGDG synthesis is carried out by MGDG synthases (MGD), which transfer a galactose residue from uridine diphosphate (UDP)-α-D-galactose (UDP-Gal) to the *sn-3* position of diacylglycerol (DAG), to form Galβ-DAG (MGDG). In cyanobacteria, MGDG is formed in two steps. The first step involves a glucosyltransferase, which transfers a glucose residue from UDP-α-D-glucose to DAG to yield Glcβ-DAG (MGlcDG) (Awai et al., 2006). In a second step, an epimerase converts the glucose moiety of MGlcDG to galactose, yielding MGDG (Awai et al., 2014). DGDG biosynthesis occurs by the same general mechanism in plants and in cyanobacteria, although using distantly related enzymes (Awai et al., 2007; Sakurai et al., 2007). The reaction is catalyzed by DGDG synthases that transfer a galactose from UDP-Gal to MGDG to produce Galα1,6Galβ-DAG (DGDG).

These gluco/galactolipid synthases belong to the large glycosyltransferase (GT) family. The transfer of the monosaccharide to the acceptor is regio- and stereo-specific. GTs can be classified as retaining or inverting enzymes depending on the stereochemical issue of the transfer reaction (i.e., retention or inversion of the anomeric configuration of the transferred sugar). Another classification has been proposed that groups GTs into families based on amino acid sequence similarities, i.e., CAZy database^1^. The same fold and reaction mechanism are expected to occur within one GT family. The database currently comprises ∼340,000 entries divided into ∼100 GT families (designated GTx, x being the family number). Despite the considerable diversity of GT sequences and function, the three-dimensional structure of GTs is remarkably conserved since only two types of folds (designated as GT-A and GT-B) have been described to date for all nucleotide sugar dependent GTs (Breton et al., 2012). The GT-A and GT-B topologies are variations around a common α/β/α scaffold, the so-called ‘Rossmann fold.’ Despite the similarities of their folds, GT-A and GT-B enzymes are unrelated and they probably evolved independently (Hashimoto et al., 2010). GTs are also characterized by an amazing conformational plasticity, which may explain their tremendous potential for accommodating a myriad of acceptor substrates (Albesa-Jové and Guerin, 2016). The gluco- and galactolipid synthases that have been described above fall into three GT families: the MGDG and DGDG synthases belong to GT28 (inverting) and GT4 (retaining), respectively, where members of both families are predicted to adopt a GT-B fold. Surprisingly, the bacterial MGlcDG synthase belongs to the inverting GT2 family, and is expected to adopt a GT-A fold. Because it is widely accepted that plastids in plants and algae originate from an ancestral cyanobacteria, the existence of two different pathways for MGDG synthesis involving two unrelated GTs raises the question of the importance of conserving MGDG in photosynthetic membranes and on the evolution history of eukaryotic MGDG synthases. The hypothesis of a lateral transfer of a MGDG synthase gene from an ancestral Chloroflexi has been proposed (Yuzawa et al., 2012).

In the past years, a number of reviews have been published covering various aspects of galactoglycerolipid metabolism and chloroplast biogenesis both in cyanobacteria and in plants (Boudière et al., 2014; Petroutsos et al., 2014; Awai, 2016; Bastien et al., 2016; Kobayashi, 2016). The present review will focus on recent developments in the biochemical and structural characterization of galactolipid synthases and of their products MGDG and DGDG, with a special focus on Arabidopsis enzymes, and how these data can be integrated in the broader context of chloroplast membrane biogenesis.

## The MGDG Synthases

The MGDG synthase activity was first ascribed to a minor membrane protein localized in the chloroplast envelope (Maréchal et al., 1994a,b). Shimojima et al. (1997) first succeeded in the cloning of the MGDG synthase from cucumber. This has led to the identification in the Arabidopsis genome of a multigenic family of MGDG synthases that were designated as type A (or MGD1) and type B (or MGD2 and MGD3) (Awai et al., 2001). The type A protein is characterized by the presence at its N-terminus of a cleavable chloroplast transit peptide of ∼100 amino acids, whereas type B displays a short addressing sequence of ∼30 residues. MGD1 is a monotopic membrane protein that was shown to localize to the outer leaflet of the inner envelope membrane (IEM) (Miège et al., 1999; Xu et al., 2005; Vojta et al., 2007). Whether the MGD1 protein faces the stromal side or the intermembrane space of the IEM is still a matter of debate. This assumption has to be revisited with the demonstration that an epimerase catalyzing the conversion of UDP-Glucose into UDP-Gal in the chloroplast stroma is essential for the biosynthesis of galactolipids in rice (Li et al., 2011). This suggests that the UDP-Gal substrate for MGD1 can be produced *in situ* in chloroplasts and that MGD1 may have access to the IEM stroma side. MGD1 was shown to be the most active isoform responsible for the synthesis of the bulk of MGDG needed for the massive expansion of thylakoids. Indeed, a MGD1 knock-out mutant (*mgd1-2*) could only grow on sucrose-supplemented media and demonstrated a complete lack of chlorophyll as well as a growth arrest after embryogenesis (Kobayashi et al., 2007).

The other Arabidopsis MGD isoforms (MGD2, MGD3) are mostly produced in non-photosynthetic tissues and, more specifically, they are induced in response to phosphate shortage (Awai et al., 2001; Kobayashi et al., 2009). In contrast to MGD1, MGD2 and MGD3 are localized in the outer leaflet of the outer envelope membrane (OEM) (Awai et al., 2001). Knock-out mutants of MGD2 and MGD3 have no striking phenotype in standard growth conditions (Kobayashi et al., 2009).

The MGD1 has been the most extensively studied galactolipid synthase. Biochemical data obtained on the purified spinach enzyme showed a sequential, random or ordered, mechanism with independent donor and acceptor binding sites (Maréchal et al., 1994a). Major advance was recently obtained in the production and crystallization of the catalytic domain of MGD1 (Rocha et al., 2013, 2016). The recombinant protein produced in *Escherichia coli* is fully active but it behaves as a monomer in solution and not as a dimer as previously proposed (Miège et al., 1999). As expected for GTs belonging to family GT28, the MGD1 protein adopts a GT-B fold (Rocha et al., 2016). The GT-B fold is characterized by two Rossmann-type domains with the catalytic site between the two domains. Perhaps the most striking feature in MGD1 was the presence of a long and flexible region of ∼50 amino acids residues in the N-terminal domain. This region seems to contribute to the anchoring MGD1 in the membrane and is essential to capture the DAG acceptor (Rocha et al., 2016). MGD1 requires anionic lipids, such as phosphatidic acid (PA) and PG for its activity (Coves et al., 1988; Dubots et al., 2010). Interestingly, it was shown that PA and PG proceed through different mechanisms, thus suggesting distinct binding sites (Dubots et al., 2010). Mutational studies indicated that PG binds to MGD1 in a region close to the DAG-binding site (Rocha et al., 2016). When tested on biomimetic langmuir monolayers, MGD1 showed a contrasted behavior toward MGDG and DGDG (Sarkis et al., 2014). The reaction product MGDG exerts a positive effect on MGD1, facilitating its binding to the membrane, whereas DGDG has a negative effect and tends to exclude the enzyme. These opposite effects illustrate the importance of the MGDG/DGDG ratio in maintaining the enzyme bound to the membrane and they also suggest that MGD1 localizes to specific microdomains (see below). Particularly, in presence of MGDG, MGD1 tends to self-organize forming elongated and reticulated lipoproteic structures (Sarkis et al., 2014). This type of organization is believed to optimize the massive production of MGDG needed for thylakoid expansion and, possibly, to contribute to the scaffolding of prothylakoids originating from the IEM (Bastien et al., 2016). MGD1 also demonstrated high affinity for DAG and PG monolayers (Sarkis et al., 2014). PG which represents ∼10% of total lipids of IEM probably plays an important role in MGD1 binding. Its role as MGD1 activator and the proximity of PG and DAG binding sites suggest that PG could help the enzyme to trap its DAG substrate. The role of PA in MGD1 activity is less clear. PA is barely detectable in chloroplast membranes but it acts as an allosteric activator of MGD1 (Dubots et al., 2010) and is the direct precursor for DAG of prokaryotic origin. A conformational change of the bilobal MGD1 enzyme may explain this allosteric effect, or alternatively, PA may induce protein dimerization. It is clear that PA plays a central role in the metabolism of lipids of photosynthetic membrane (Dubots et al., 2012).

## The DGDG Synthases

In Arabidopsis, two genes coding for DGDG synthases have been identified and characterized (Dörmann et al., 1999; Kelly and Dörmann, 2002). DGD1 is responsible for the synthesis of the bulk of DGDG (> 90%) in chloroplasts under normal growth conditions, whereas DGD2 only produces minor amounts of DGDG (Kelly et al., 2003). Only the part of MGDG with a eukaryotic signature is used by DGDG synthases to form DGDG. A *dgd1* mutant shows severe dwarfism and loss of photosynthesis efficiency (Dörmann et al., 1995), whereas *dgd2* mutants were much less affected (Kelly et al., 2003). Although the expression of both DGD1 and DGD2 is induced during phosphate deprivation, DGD2 seems to be the major enzyme to provide DGDG for the extraplastidial membranes (Härtel et al., 2000; Kelly and Dörmann, 2002). Both enzymes are localized to the OEM (Froehlich et al., 2001; Kelly et al., 2003), with their catalytic domains oriented to the cytosolic side, thus raising the question of the trafficking of their substrate MGDG synthesized in the IEM. DGD1 seems to use MGDG formed by MGD1 in the IEM whereas DGD2, under Pi shortage, preferably uses MGDG generated by MGD2/MGD3 in the OEM (Benning, 2009).

The catalytic domain of DGDG synthases is predicted to adopt a similar GT-B fold as MGD1. DGD1 has a unique feature that is not observed in DGD2. The catalytic domain is preceded by a large N-terminal region comprising about 330 amino acids. This domain does not contribute to the galactosyltransferase reaction but is required for insertion of DGD1 into the OEM (Froehlich et al., 2001). Very recently, the role of this N-terminal extension (N-DGD1), predicted all-α with coiled-coil domains, was addressed (Kelly et al., 2016). Using a series of chimeric constructs to complement a *dgd1* mutant, the authors demonstrated that N-DGD1 was essential for enabling galactolipid transfer between envelope membranes. Also, N-DGD1 mediates a PA-dependent membrane fusion *in vitro*. These data represent a breakthrough in our understanding of MGDG and DGDG trafficking between IEM and OEM. In the case of DGD2, it was proposed that the enzyme interacts with the membrane through its N-terminal domain, via interactions with negatively charged lipids (Szpryngiel et al., 2011), but also with part of its C-terminal domain which could act as a lipid sensing switch (Szpryngiel and Mäler, 2016).

Another pathway for DGDG synthesis is mediated by the GGGT, an enzyme that transfers a galactose from one molecule of MGDG to another MGDG with concomitant release of a DAG moiety (Moellering and Benning, 2011). However, it must be noted that DGDG produced is different with respect to the glycosidic linkage formed (ββ-DGDG instead of αβ-DGDG). Although the GGGT enzyme was found localized to the OEM, it does not contribute to the net synthesis of galactolipids in normal growth conditions and during Pi deprivation (Kelly et al., 2003). GGGT also demonstrates a processive activity leading to the formation of tri- and tetra-galactolipids. The role of GGGT has been enigmatic for many years. Recently, this GGGT was shown to be involved in freezing tolerance (Moellering et al., 2010). The GGGT encoding gene has been identified as SENSITIVE TO FREEZING 2 (SFR2) in Arabidopsis. SFR2 is not vital for normal growth and development but the freezing sensitivity of the *sfr2* mutant suggested that the enzyme was involved in lipid remodeling of chloroplast membranes. Changing the ratio of bilayer-/non-bilayer-forming lipids to favor the formation of lamellar bilayers is a mechanism to stabilize membranes during freezing stress. One can reasonably speculate a role of SFR2 as a sensor of biophysical and compositional changes in membranes, triggering lipid remodeling in response to abiotic stress.

## Roles of Galactolipids in the Structure and Biogenesis of Thylakoids

The lipid composition of chloroplast membranes has been remarkably conserved through evolution from cyanobacteria to plants (Petroutsos et al., 2014). Particularly the MGDG/DGDG ratio appears to be highly stable (Boudière et al., 2014). The hypothesis of a specific contribution of galactolipids in the organization of thylakoid membranes as stacked flattened cisternae that can pile up to form grana was steadily addressed. This type of organization appears crucial to ensure enough density of photosystems to capture light energy. By reconstituting membranes made of natural thylakoid lipid extracts, it was found that the lipid mixture can self-organize as a membrane bilayer and can reversibly switch from the hexagonal II (HII) to the lamellar (Lα) phase (Demé et al., 2014). The transition can be fine-tuned by the lipid composition, particularly the MGDG/DGDG ratio, and hydration. These studies highlighted the critical role of the bilayer-forming DGDG, described as a galactolipid zipper (Demé et al., 2014), in membrane stacking via hydrogen bonds between polar heads of adjacent bilayers. These interactions also contribute to balance the repulsive electrostatic effects of the anionic lipids PG and SQDG, thus favoring grana stacking (Demé et al., 2014; Kanduč et al., 2017). The biophysical properties of galactolipids could explain the conservation of thylakoid membrane composition throughout evolution.

Thylakoid biogenesis results in etioplasts from light activation of the cubic phase tubular structure of prolamellar bodies where a critical level of MGDG is required for the formation of the photoactive protochlorophyllide-LPOR-NADPH complex and its oligomerization (Fujii et al., 2017; Gabruk et al., 2017). Alternatively, in most types of plastids, thylakoid biogenesis results also from formation of *de novo* structures from the IEM. Different scenarios have been proposed for formation from IEM, including the budding of vesicles or flattened invaginations from the IEM and HII intermediate structures (Bastien et al., 2016). The unique localization of MGD1 in the IEM and the propensity of MGDG to self-organize in inverse micelles (HII) rather than in bilayers are important clues in the context of thylakoid biogenesis. A HII↔Lα phase transition triggered by subtle variations in the MGDG/DGDG ratio, as a result of favored or disfavored MGD1 binding to the IEM, can be a driving force that governs thylakoid membrane expansion. To support this assumption, membrane connections were observed between the IEM and thylakoids in the severe genetic background of *mgd1-2* mutant (Kobayashi et al., 2007), and in wild-type Arabidopsis plants treated with galvestin-1, an inhibitor of MGDG synthases (Botté et al., 2011). Based on these observations, a non-lamellar/non-vesicular model has been proposed in which the transit of lipids between the IEM and thylakoids operates, at least in the early stage of thylakoid biogenesis, through HII regions enriched in MGD1-MGDG (Bastien et al., 2016). This model does not exclude the contribution of other cellular partners such as IM30/VIPP1, a protein triggering membrane fusion in chloroplasts, and which was shown to bind to anionic lipids (i.e., PG and SQDG) (Hennig et al., 2015).

**FIGURE 2:**
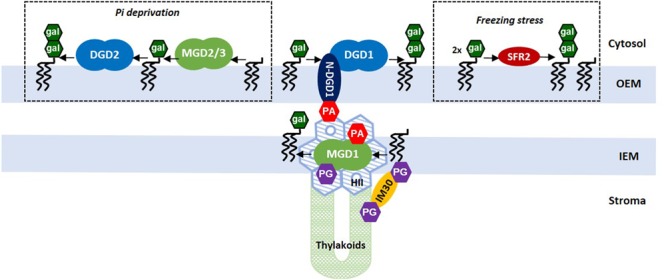
Biosynthesis of galactolipids in the chloroplast envelope membranes in Arabidopsis. The three enzyme systems are represented. The main pathway is mediated by MGD1 and DGD1 which provide the bulk of galactolipids for all chloroplast membranes in normal growth conditions. MGD2/3 and DGD2 are activated in response to Pi deprivation, and SFR2 is activated in response to a freezing stress. Flux of galactolipids between IEM and OEM are facilitated by PA-mediated contacts with N-DGD1 (as proposed by Kelly et al., 2016). The MGD1-MGDG association in the IEM forms a HII platform that allows the transit of galactolipids to newly synthesized thylakoids through a non-lamellar/non-vesicular process (as proposed by Bastien et al., 2016). This process might involve other cellular partners such as the IM30/VIPP1 protein (Hennig et al., 2015). Can MGD1, inserted in a locally non-lamellar microdomain, have access on one side or the other side of the IEM, or even both, has yet to be determined.

This review highlights the unique and irreplaceable roles of MGDG and DGDG, which have very contrasted biophysical properties, in the biogenesis and architecture of chloroplast membranes. **Figure 2** gives an overview of our current knowledge on galactolipid synthases, particularly their localization in the chloroplast envelope, and highlights the central roles of MGD1 and DGD1 in regulating the flux of galactolipids between IEM and OEM, and between IEM and thylakoid membranes. The fine-tuning of these enzymes, by controlling HII↔Lα phase transitions, appears important for nascent thylakoid development, for membrane–membrane interactions and the development of flattened cisternae. This also raises the major but still unresolved question of the coexistence of HII+Lα phases. In particular, the location and regulation of MGDG-rich HII regions, required for the functional violaxanthin/asthaxanthin cycle (Goss et al., 2017), and the relation with domains containing the photosystems, is clearly a major puzzling question for the future.

## Author Contributions

The subject was the result of a fruitful collaboration involving all co-authors. JR, MN, AG-E, JJ, EM, MB, and CB agreed to contribute this review and participated in delineating the content of the topic, and in the writing and preparation of figures.

## Conflict of Interest Statement

The authors declare that the research was conducted in the absence of any commercial or financial relationships that could be construed as a potential conflict of interest.

## Footnotes

1 http://www.cazy.org/

## Abbreviations

DGDG: digalactosyldiacylglycerol
IEM: inner envelope membrane
MGDG: monogalactosyldiacylglycerol
OEM: outer envelope membrane
PA: phosphatidic acid
PG: phosphatidylglycerol
SQDG: sulfoquinovosyl diacylglycerol
